# Estimating Soil Moisture Distributions across Small Farm Fields with ALOS/PALSAR

**DOI:** 10.1155/2016/4203783

**Published:** 2016-07-26

**Authors:** Yuki Kojima, Kazuo Oki, Kosuke Noborio, Masaru Mizoguchi

**Affiliations:** ^1^Faculty of Engineering, Gifu University, 1-1 Yanagodo, Gifu City, Gifu 501-1193, Japan; ^2^Institute of Industrial Sciences, The University of Tokyo, 4-6-1 Komaba, Meguro-ku, Tokyo 153-8505, Japan; ^3^School of Agriculture, Meiji University, 1-1-1 Higashimita, Tama-ku, Kawasaki City, Kanagawa 214-8571, Japan; ^4^Graduate School of Agricultural and Life Sciences, The University of Tokyo, 1-1-1 Yayoi, Bunkyo-ku, Tokyo 113-8657, Japan

## Abstract

The ALOS (advanced land observing satellite) has an active microwave sensor, PALSAR (phased array L-band synthetic aperture radar), which has a fine resolution of 6.5 m. Because of the fine resolution, PALSAR provides the possibility of estimating soil moisture distributions in small farmlands. Making such small-scale estimates has not been available with traditional satellite remote sensing techniques. In this study, the relationship between microwave backscattering coefficient (*σ*) measured with PALSAR and ground-based soil moisture was determined to investigate the performance of PALSAR for estimating soil moisture distribution in a small-scale farmland. On the ground at a cabbage field in Japan in 2008, the soil moisture distribution of multiple soil layers was measured using time domain reflectometry when the ALOS flew over the field. Soil moisture in the 0–20 cm soil layer showed the largest correlation coefficient with *σ* (*r* = 0.403). The *σ* values also showed a strong correlation with the ground surface coverage ratio by cabbage plants. Our results suggested that PALSAR could estimate soil moisture distribution of the 0–20 cm soil layer across a bare field and a crop coverage ratio when crops were planted.

## 1. Introduction

Based on the 10-year Global Earth Observation System of Systems (GEOSS) implementation plan, integration of global earth observation data associated with climate change and hydrological cycle has been conducted with the cooperation of nearly 60 countries [1]. Because water quantity in soils plays an important role in controlling energy balance and evapotranspiration rates on the earth surface [2, 3], the importance of estimating soil moisture distribution on the earth surface with satellite remote sensing has been recognized [4]. For example, Njoku et al. [5] and Koike et al. [6] developed algorithms to estimate soil moisture content with microwave sensors equipped in satellites and evaluated the applicability of their algorithms by comparing the estimates and ground measurements.

Microwave sensor for satellite remote sensing is categorized into three methods: (1) synthetic aperture radar (SAR) (active microwave method), (2) microwave radiometer (passive microwave method), and (3) the combination of active and passive methods to estimate surface soil moisture [7]. The SAR and microwave radiometer have different sampling scale of the soil moisture estimates. While the microwave radiometer covers only large area (more than 10 km), the SAR has a finer sampling scale (approximately 20 m) [8]. The microwave radiometer has been more applied because of its advantages of high temporal resolution and less influence of surface conditions [9]. And recent study succeeded in enhancing the spatial resolution of radiometer as fine as 1 km [10]. Although the SAR has fine spatial resolution, the current temporal resolution is not fine enough for the hydrological studies and the SAR is sensitive to the surface roughness [11, 12]. Despite the disadvantages, the benefit of estimating fine scale (meter order) soil moisture distribution is attractive for soil and plant scientists, irrigation engineers, hydrologist, agronomists, and farmers.

An advanced land observing satellite (ALOS), which was named DAICHI, was launched on January 24, 2006, by Japan Aerospace Exploration Agency (JAXA) (the ALOS operation has ended on May 2011 due to the power loss). The ALOS embedded a phased array L-band synthetic aperture radar (PALSAR) that enables us to observe the earth surface without restriction made by weather or solar radiation. Microwaves used in the PALSAR are categorized into a horizontal polarized wave (H) and a vertical polarized wave (V) depending on the direction of the electric field toward the earth surface. Since either the horizontal or the vertical polarized wave can be selected when the radar sends and receives both the microwaves, the PALSAR utilizes information from the four combinations of polarized waves (HH, HV, VV, and VH) [13]. When a single polarization mode is used, that is, HH or VV, the PALSAR has the finest resolution up to 6.5 m × 6.5 m at minimum [14]. In addition, L-band microwave used in the PALSAR has a long wavelength (23 cm) meaning the microwave penetrates plant canopies so that soil moisture is expected to be measured with no influence of plant conditions on the soil surface [15].

Because the PALSAR targets a small area using the single polarization mode, soil moisture estimation with PALSAR may be of interest especially for farmers in Japan. Farmland size in Japan is generally smaller than those in other countries. For example, the average farmland size in the United States is approximately 450 ha [16] while that in Japan is 1.5 ha [17]. The spatial variabilities of soil moisture, infiltration rate, and other soil properties are found even in a small area [18, 19]. Understanding the spatial variability of soil moisture in a small farmland may help Japanese farmers detect patches where crops suffer from water stress to determine irrigating areas for stable crop production. The PALSAR has a potential to evaluate the spatial variability of soil moisture in a small farmland whereas traditional satellite remote sensing sensors did not provide fine enough resolution. Previous studies using the PALSAR single polarization mode to estimate soil moisture were more or less focused on relatively large areas such as that by Susaki [20] estimating soil moisture distribution in noninundated paddy fields with the mesh size of 100 m and that by Sonobe et al. [21] evaluating the averaged soil moisture of an entire farmland. To date, however, little attention has been paid to use the smallest PALSAR mesh size (6.25 m) when the single polarization mode is used to investigate soil moisture distribution. The previous studies also have no interests in the vertical distribution of soil moisture. Volumetric soil water content in the field is well known as functions of both space and depth. Njoku and Entekhabi [22] showed that the sampling depth of remote sensing microwave, that is, penetration of microwave, varied with soil moisture. For example, L-band microwave penetrates 1 m deep in dry soil but only 0.1 m deep in wet soil in their study. None of the previous studies investigated the penetration depth of the PALSAR with the single polarization mode.

Therefore, to obtain a fundamental knowledge for estimating soil moisture with the PALSAR, we aimed in this study to establish a relationship between backscattering coefficients measured with the single polarization mode of PALSAR and volumetric water content, in small-scale farmlands. The backscattering coefficient indicates the degree of microwave reflected from the surface and received by a radar receiver in a unit area [23]. The backscattering coefficient is usually expressed with the unit of dB, and it depends on various surface conditions such as soil moisture, surface roughness, and surface coverage [24]. Using average volumetric water content measured with the various depths of TDR probes, we investigated how deep microwave from the PALSAR single polarization mode penetrated in soil. In addition to the volumetric water content, a relationship between backscattering coefficients and surface crop coverage was investigated to better understand the PALSAR application.

## 2. Relationship between Microwave Reflection and Soil Moisture

The PALSAR sends microwaves from its antenna, and with the antenna it receives the microwaves reflected from the earth surface. Microwave propagating in the air reflects when electrically heterogeneous materials exist in its pathway. Thus, when the microwave reaches the soil surface, considered as an electrically heterogeneous material, it reflects back to the antenna. The degree of the reflection may be described as follows [25]:

(1) $$\begin{array}{c} \Gamma =\frac{\sqrt{\varepsilon_{a}}-\sqrt{\varepsilon_{s}}}{\sqrt{\varepsilon_{a}}+\sqrt{\varepsilon_{s}}}, \end{array}$$

where Γ is a reflection coefficient, *ε*_*a*_ is the dielectric constant of atmosphere, and *ε*_*s*_ is the dielectric constant of soil surface. The value for *ε*_*a*_ is approximately 1, and the value for *ε*_*s*_ ranges approximately between 3 and 40 [26]. The magnitude of reflection is weak when the absolute value of Γ is close to 0, and it is strong when Γ is close to 1. Based on (1), the absolute value of Γ increases as *ε*_*s*_ increases. Because *ε* of water (approximately 80) is much higher than those of other soil constituents, that is, *ε* of air is 1 and *ε* of soil particles is between 2 and 12, *ε*_*s*_ strongly depends on soil moisture [27]. This is the principle of estimating soil moisture with microwaves sent from the PALSAR. The PALSAR provides imagery with 6.5 m × 6.5 m meshes in which each mesh has its own brightness (DN: digital number) associated with microwave reflection at the surface. The backscattering coefficient *σ* (dB) may be calculated from DN as follows [28]:

(2) $$\begin{array}{c} \sigma =20\log_{10}\left(\;DN\;\right)-83. \end{array}$$

## 3. Materials and Methods

### 3.1. Backscattering Coefficient and Surface Coverage

On July 3, 2008, the degree of surface crop coverages was determined at 5 cabbage fields in a cool high-mountain region in Tsumagoi Village, Agatsuma-gun, Gunma Prefecture in central Japan, at the similar time when the ALOS passed over the fields. The terrestrial coordinates of each cabbage field were determined with GPS (Table 1). In each field, surface images were taken with a digital camera. The collected surface images were analyzed with binary image processing and surface coverage was calculated. From the PALSAR image (off-Nadir angle: 34.3, ascending and polarimetric mode), brightness of each site was extracted and converted into the backscattering coefficient. The polarized waves of the PALSAR on July 3, 2008, were HH and HV. The correlation between the measured surface coverage and backscattering coefficient was determined.

### 3.2. Backscattering Coefficient and Volumetric Water Content

**Figure 1:**
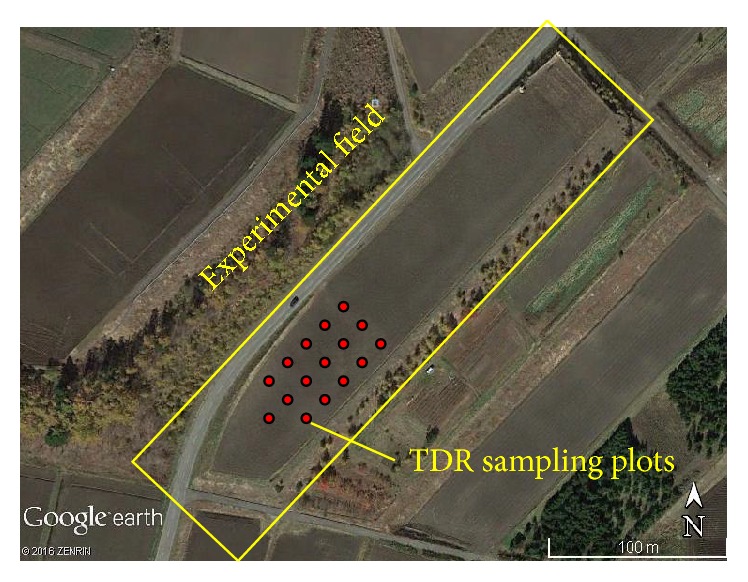
Layout of plots where TDR soil moisture measurements were conducted in an experimental field on August 18, 2008 (information added on Google Earth image).

**Figure 2:**
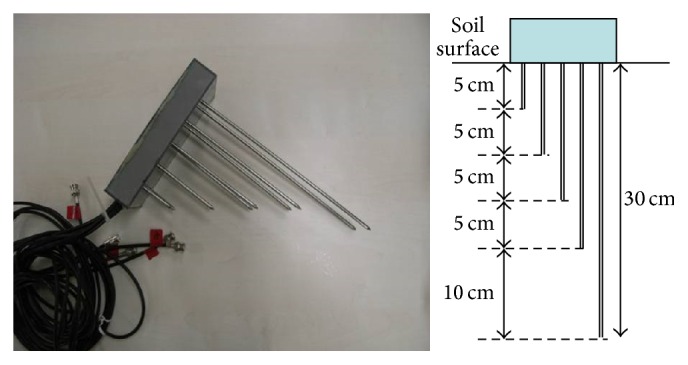
Developed TDR multiprobes to measure volumetric water content at multiple soil layers.

On August 18, 2008, volumetric water contents at 16 spots in a bare cabbage field (36°29′45.60′′ N, 138°27′36.71′′ E) were measured correspondingly to the PALSAR sending time. The spots location is shown in Figure 1. The measurement of volumetric water contents was made with time domain reflectometry (TDR). The detail of TDR techniques is well described in [27]. The TDR multiprobes consist of five pairs that are 5 cm, 10 cm, 15 cm, 20 cm, and 30 cm long with 5 mm dia. stainless-steel rods with 45 mm spacing between the rods of a pair were developed (Figure 2). By vertically inserting the TDR probes from the soil surface, average volumetric water contents at 0–5 cm, 0–10 cm, 0–15 cm, 0–20 cm, and 0–30 cm soil layers were measured. The terrestrial coordinates of the 16 spots were determined with GPS. The backscattering coefficients of the spots were calculated with imagery from the PALSAR (off-Nadir angle: 34.3, ascending and single polarization mode). The polarized wave of the PALSAR on August 18 was HH. The field had a consistent slope with a homogeneously smooth surface so that we assumed that the influence of slope and surface roughness on backscattering coefficient was negligible. The correlation between backscattering coefficient and volumetric water contents at each soil layer was determined.

## 4. Results and Discussion

### 4.1. Relationship between Backscattering Coefficient and Surface Coverage

**Figure 3:**
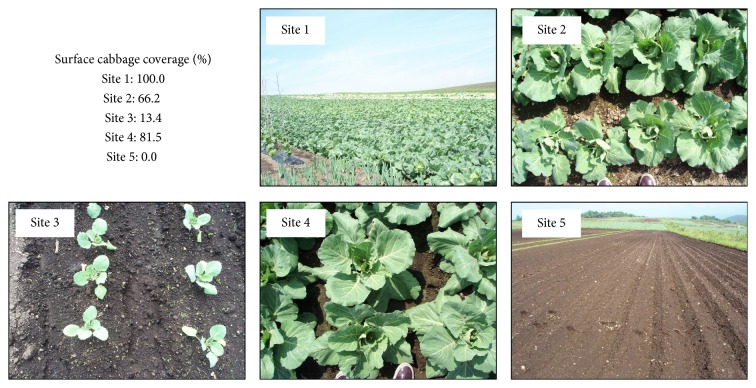
Calculated surface coverage rates and images of various cabbage growth stages at each site.

**Figure 4:**
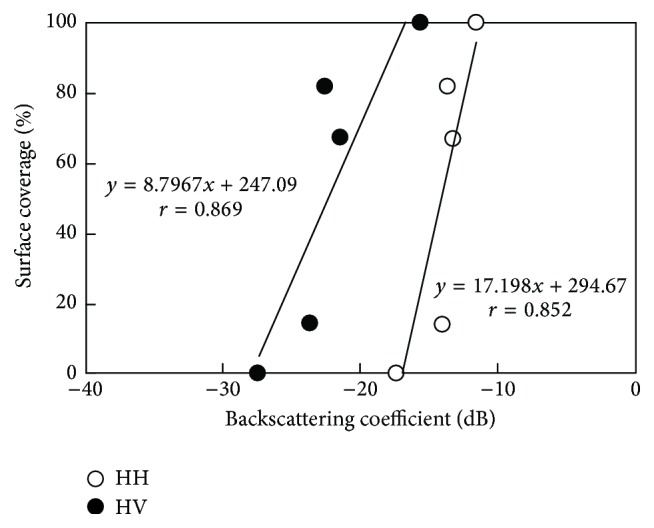
The relationship between surface cabbage coverage and backscattering coefficient.

Figure 3 shows the field images taken with the digital camera and surface coverage determined with the binary image processing. The crop coverage of site 1 and site 5 were visually determined as 100% and 0%, respectively.  Figure 4 shows the relationship between the surface crop coverage and backscattering coefficient. Although the sample number is small, there was a significant correlation between the crop coverage and backscattering coefficient with both HH and HV. The correlation coefficient with HV (*r* = 0.869) was slightly higher than that with HH (*r* = 0.852). This may partly be resulting from the microwave reflected by water retained in cabbage plants. Even though the PALSAR uses L-band microwave, plants on the surface affect backscattering coefficient. As shown in Figure 4, the PALSAR successfully detected the crop coverage with a small mesh size. Detecting various crop growing stages based on crop coverage would be possible for small-scale farmlands.

### 4.2. Relationship between Backscattering Coefficient and Volumetric Water Content

**Figure 5:**
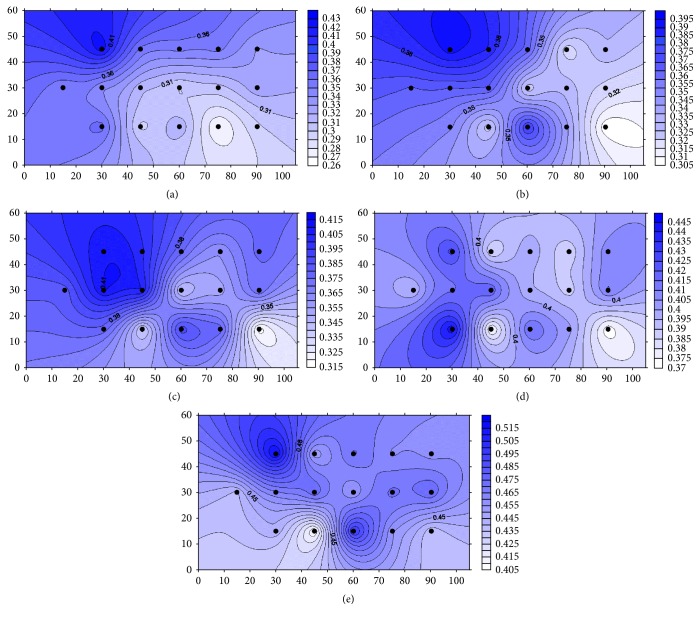
Spatial distributions of volumetric water content estimated from TDR measurements at (a) 0–5 cm, (b) 0–10 cm, (c) 0–15 cm, (d) 0–20 cm, and (e) 0–30 cm soil layers.

Based on volumetric water contents measured with TDR, volumetric water content distributions at 0–5 cm, 0–10 cm, 0–15 cm, 0–20 cm, and 0–30 cm soil layers were estimated, and isolines were drawn in Figure 5 with Surfer ver. 10 (Golden Software Inc., Golden, CO). Volumetric water content increased as a sampling depth increased. Volumetric water content was spatially distributed between 0.27 and 0.43 m^3^ m^−3^ at 0–5 cm, between 0.31 and 0.39 m^3^ m^−3^ at 0–10 cm, between 0.32 and 0.41 m^3^ m^−3^ at 0–15 cm, between 0.37 and 0.44 m^3^ m^−3^ at 0–20 cm, and between 0.41 and 0.52 m^3^ m^−3^ at 0–30 cm soil layers. Volumetric water contents at the 0–5 cm, 0–10 cm, and 0–15 cm soil layers in Figures 5(a), 5(b), and 5(c), respectively, were relatively higher in the southwest (lower slope) of the field, indicated at around the upper left corner in Figure 5(a)–5(e), than that in the northeast (upper slope) since the field sloped down to the southwest from the northeast (Figure 1). Volumetric water content at 0–20 cm and 0–30 cm soil layers showed complicated distribution; that is, wet areas and dry areas were mixed (Figures 5(d) and 5(e)).

**Figure 6:**
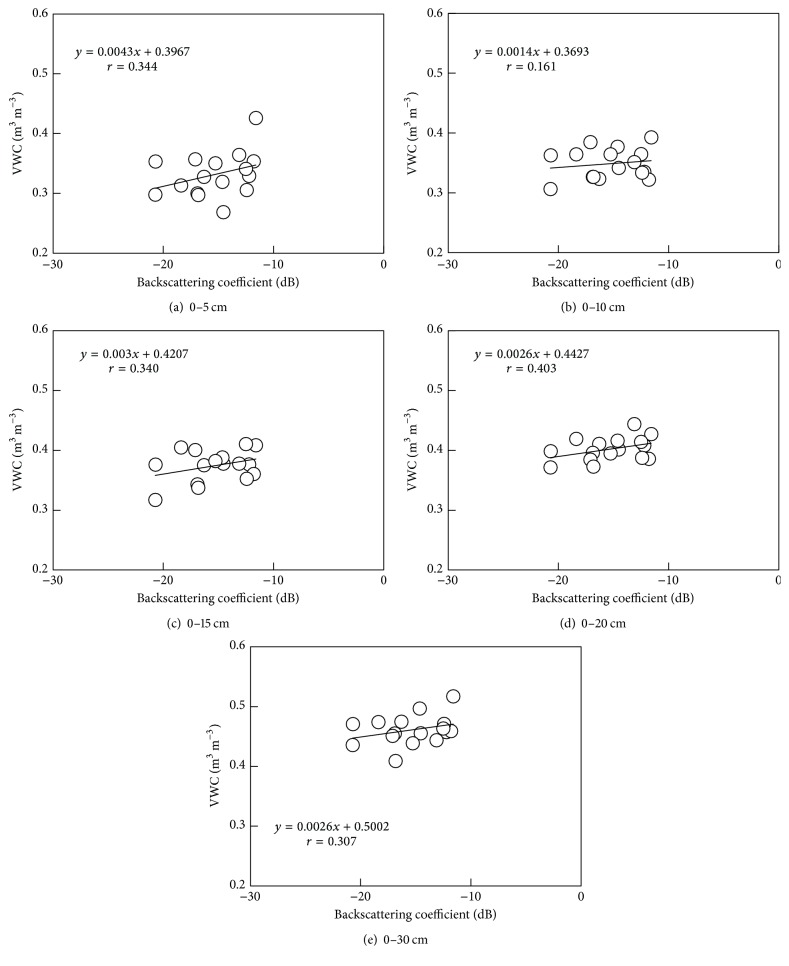
Relationship between backscattering coefficient and volumetric water content (VWC) measured with the TDR method at (a) 0–5 cm, (b) 0–10 cm, (c) 0–15 cm, (d) 0–20 cm, and (e) 0–30 cm soil layers.

Figure 6 shows a relationship between volumetric water content at each soil layer and PALSAR backscattering coefficient. Correlations between volumetric water content and backscattering coefficient were not very strong for all the layers. The largest correlation coefficient (*r* = 0.403) found for the 0–20 cm soil layer implied that the PALSAR microwave penetrated up to 20 cm deep in soil. Field measurements of volumetric water content on August 18, 2008, mostly ranged between 0.30 and 0.50 m^3^ m^−3^, a wetter range. Adding a dryer range of volumetric water content, for example, 0.05–0.3 m^3^ m^−3^, may provide better correlations between volumetric water content and backscattering coefficient.

**Figure 7:**
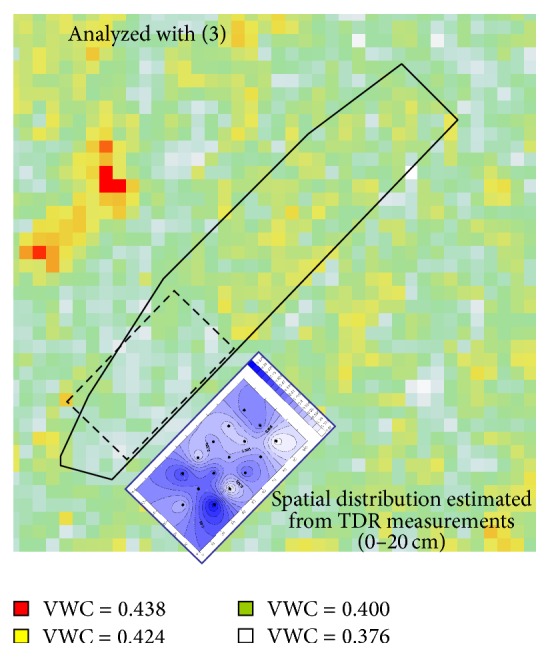
Spatial distribution of volumetric water content (VWC) estimated with PALSAR imagery using (3).

A mosaic map showing volumetric water content distribution in Figure 7 was made from PALSAR imagery using a linear regression equation with the largest *r* value shown in Figure 6(d). The equation is read as

(3) $$\begin{array}{c} \theta =0.0026\sigma +0.4427, \end{array}$$

where *σ* is the backscattering coefficient (dB) defined by (2). Distributions of volumetric water content made with PALSAR using (3) and those made with TDR measurement at 0–20 cm soil layer seem to agree better with each other (Figure 7). The upper half of the field shown in Figure 7 (the outside area of the broken line) was covered with cabbage plants at the PALSAR sending time. A cabbage-covered area obviously showed different values of the backscattering coefficient from those of the bare field. The backscattering coefficients varied even within the cabbage-covered area as well as in the bare field. This implies that the PALSAR detected various water contents of cabbage plants or a coverage ratio as shown in Figures 3 and 4. Conclusively, the PALSAR has a potential to measure soil moisture distribution in bare small fields and to measure various crop growth stages when the field is under cultivation of crops.

## 5. Conclusions

We investigated how well the AOS/PALSAR estimated soil moisture distribution in a small-scale farmland. There was a linear correlation between volumetric water content and PALSAR backscattering coefficient when the soil surface was bare. The PALSAR seemed to detect volumetric water content at the 0–20 cm soil layer. When crops were cultivated in the field, the PALSAR was able to measure a crop coverage ratio as well.

In this study, correlations between volumetric water content and backscattering coefficient were not very strong. The assumption we made that the variability of those factors was negligible for the PALSAR measurement because of the steady and homogeneous slope and surface roughness might be inaccurate. The correction of backscattering coefficient corresponding for various slope and surface roughness may improve estimates of volumetric water content as reported by others [20, 21]. It is well known that volumetric water content spatially varies even within a short distance such as 10 m [29]. The PALSAR measures average volumetric water content inside a 6.5 × 6.5 m mesh area [30] whereas TDR only measures volumetric water content of a pinpoint area. Representative element volume (REV) differing from one measuring technique to another and the nature of spatially varied volumetric water content may be responsible for insignificant correlation coefficients between volumetric water content and backscattering coefficient. Since the sampling areas of TDR and PASAR substantially differ, a similar sampling area to PALSAR with such a ground penetrating radar [31] may be preferred for the ground-based soil moisture measurement.

## Figures and Tables

**Table 1:** Locations of cabbage fields for the coverage experiment on July 3, 2008.

	North	East
Site 1	36°29′45.60′′	138°28′40.61′′
Site 2	36°30′06.94′′	138°28′26.29′′
Site 3	36°30′55.45′′	138°27′59.61′′
Site 4	36°31′00.85′′	138°28′34.68′′
Site 5	36°31′26.74′′	138°28′21.91′′
